# Supplementary material for: Ensemble dimensionality reduction and feature gene extraction for single-cell RNA-seq data
Source: Nat Commun. 2020 Nov 17;11:5853. doi: 10.1038/s41467-020-19465-7 (PMC7673125; doi:10.1038/s41467-020-19465-7)
Supplement: Supplementary file 1 — Supplementary Information [file 41467_2020_19465_MOESM1_ESM.pdf]

Supplementary Information for Ensemble Dimensionality Reduction and Feature Gene Extraction for Single-cell RNA-seq Data

Sun et al.

Supplementary notes

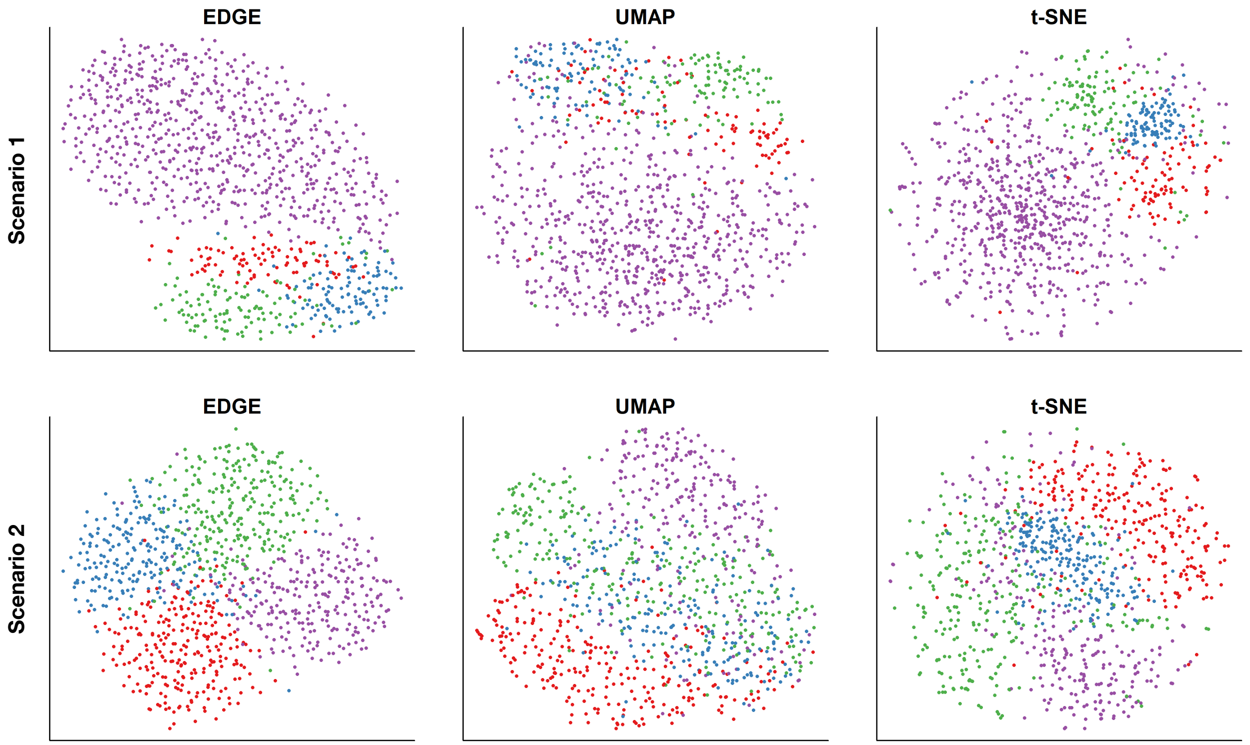

Supplementary Figure 1 Embedding results of EDGE, UMAP, and t-SNE in Scenario 1 and 2 of simulation studies.

Additional simulation studies. In the following two scenarios, we let the numbers of cells and genes be $10,000$ and 500, and the proportion of differentially expressed (DE) genes be 35%. We generated 100 simulated datasets using Splatter. The performance of EDGE, together with t-SNE and UMAP, was measured by the prediction accuracy of the rare population through out-of-bag (OOB) prediction errors in random forests over 100 simulation replicates.

Supplementary Scenario 1. We designed this scenario to study the minimal population percentage that can be detected by EDGE. The total number of cell types was 5, among which one was a rare cell type with the percentage ranging from 0.1% to 5%. The other four major cell types were set to have equal proportions. For settings with different rare cell percentages, EDGE achieved the highest prediction accuracy on average (Supplementary Figure 2). When the rare population percentage was greater than 0.5%, EDGE obtained a prediction accuracy above 80% on average. To explore the performance of EDGE in detecting multiple rare populations, we further let the number of rare populations be three, and all

other settings remained the same. EDGE ranked first in terms of prediction accuracy in all three rare populations (Supplementary Figure 3).

Supplementary Scenario 2. In this scenario, we investigated the prediction accuracy of the rare cell type when the total number of cell types changed from 2 to 10. Among all the cell types, one was the rare population with a percentage of 1%. The major cell types had equal proportions. Across all settings, EDGE maintained the highest prediction accuracy on average (Supplementary Figure 4). We further increased the number of rare populations to three, and the total number of cell types changed from 4 to 10. All other settings remained the same. EDGE outperformed other methods in all three rare populations (Supplementary Figure 5).

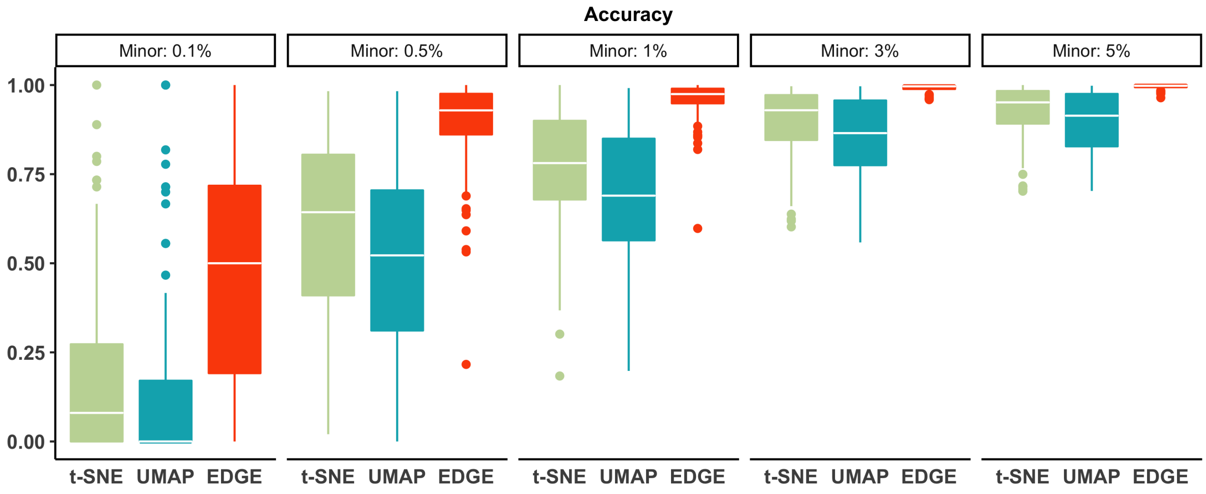

Supplementary Figure 2 The accuracy of random forests classifiers in predicting the labels of a rare cell type using the learnt embeddings as input. The percentage of rare population varies from 0.1% to 5%, and the results are summarized from 100 simulation runs. In the boxplots, we show the median (central lines), first and third quartile (box limits), and the whiskers extended to the lowest and highest points within 1.5 interquartile range of the first and third quartiles, respectively.

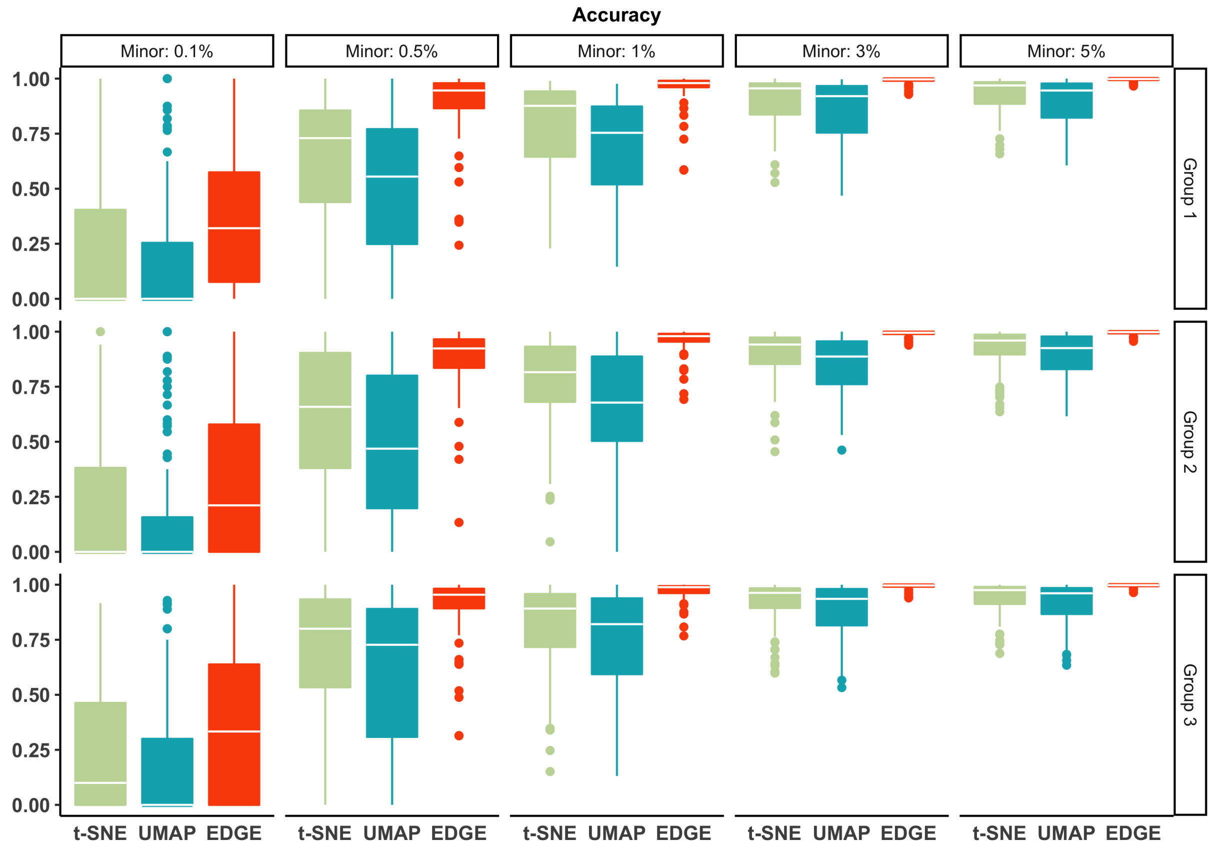

Supplementary Figure 3 The accuracy of random forests classifiers in predicting the labels of three rare cell groups using the learnt embeddings as input. The percentage of each rare cell group varies from 0.1% to 5%, and the results are summarized from 100 simulation runs. In the boxplots, we show the median (central lines), first and third quartile (box limits), and the whiskers extended to the lowest and highest points within 1.5 interquartile range of the first and third quartiles, respectively.

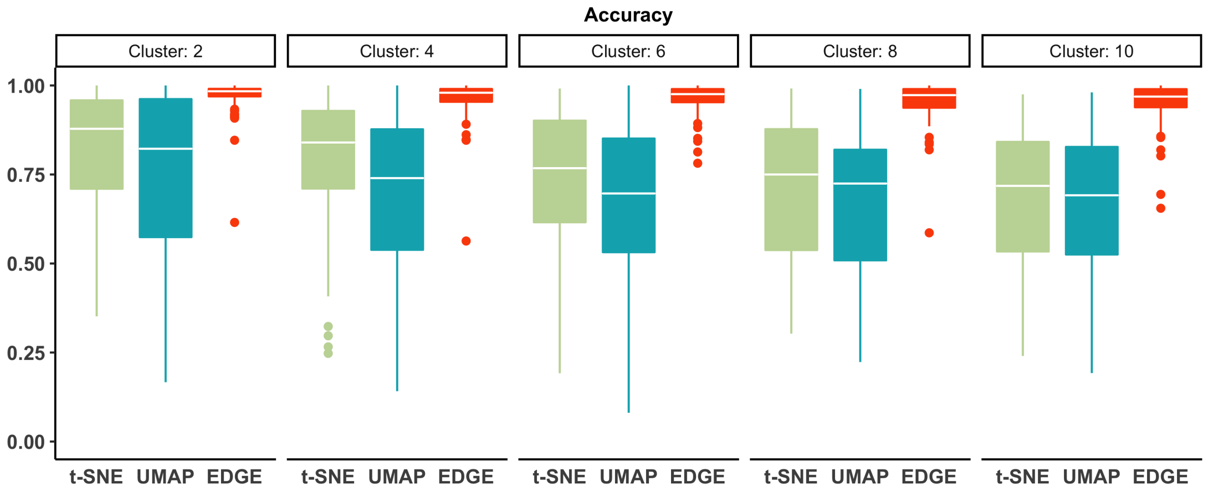

Supplementary Figure 4 The accuracy of random forests classifiers in predicting the labels of a rare cell type using the learnt embeddings as input. The total number of cell types varies from 2 to 10, and the results are summarized from 100 simulation runs. In the boxplots, we show the median (central lines), first and third quartile (box limits), and the whiskers extended to the lowest and highest points within 1.5 interquartile range of the first and third quartiles, respectively.

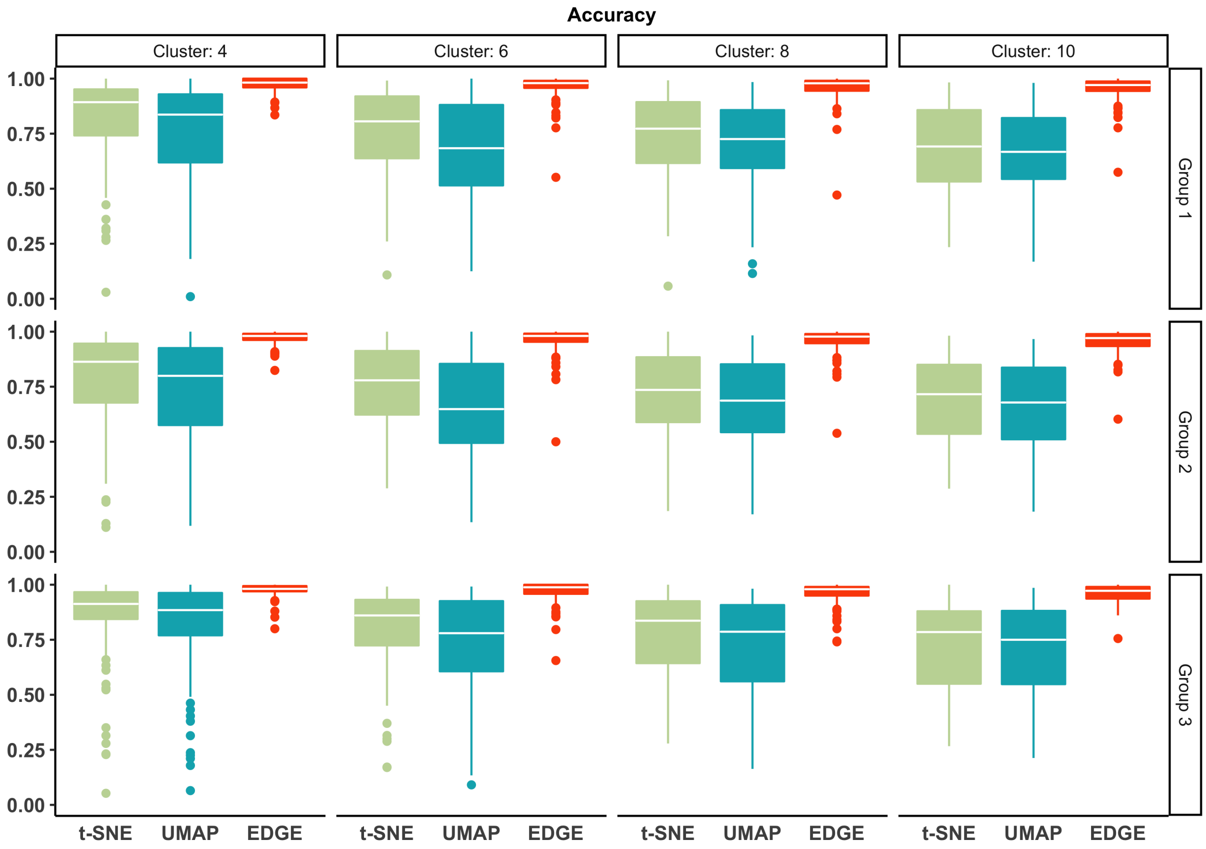

Supplementary Figure 5 The accuracy of random forests classifiers in predicting the labels of three rare cell groups using the learnt embeddings as input. The total number of cell types varies from 4 to 10, and the results are summarized from 100 simulation runs. In the boxplots, we show the median (central lines), first and third quartile (box limits), and the whiskers extended to the lowest and highest points within 1.5 interquartile range of the first and third quartiles, respectively.

Feature genes identification in real datasets. The violin plots of feature genes identified from the Jurkat and mouse brain scRNA-seq datasets are displayed here. In the Jurkat dataset, EDGE identified 17 feature genes. Each cell type was found to have a group of genes that displayed up-regulated expression patterns (Supplementary Figure 6). For example, *CD1E* and *CD3D* were highly expressed in Jurkat cells, while *HAND1*, *GADD45B*, and *BAMBI* were up-regulated in 293T cells. Furthermore, some top-ranked genes were marker genes for T cells. For instance, the *CD3D* gene engaged in T-cell development and signal transduction [1]. In the mouse brain dataset, EDGE detected 43 feature genes (Supplementary Figure 7). The genes, *CCL2* and *CCL7*, known as the two most common activators of microglia during the process of developing neuropathic pain [2], were ranked as top feature genes and only up-regulated in microglia cells. Another three genes, *FCGR1, FCRLS, C1QC*, were top enriched genes in microglia cells [3]. Among the feature genes for interneurons, *GAD1* and *HTR3A* were known marker genes for interneuron and its subclasses [4]. Astrocytes-Ependymal cells were featured by *PRDX6, CNP*, and *ACSBG1*. The gene *PRDX6* was found primarily in astrocytes cells [5, 6], and the gene *ACSBG1* was a known marker for astrocytes cells [7].

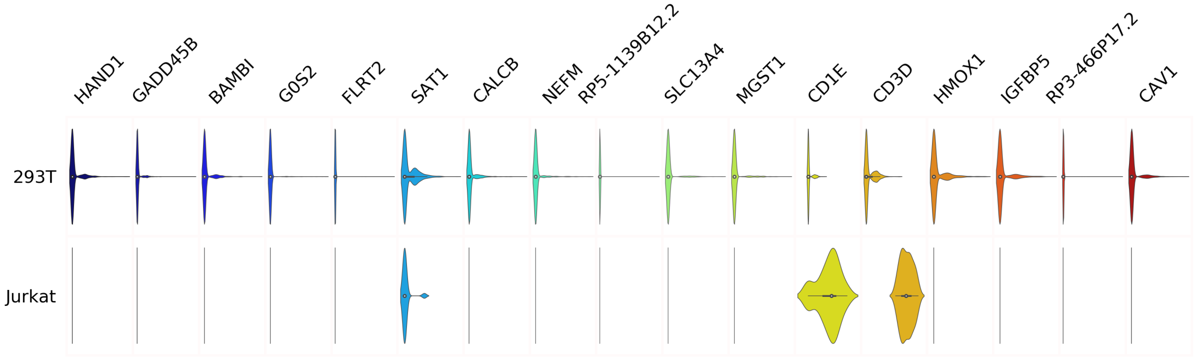

Supplementary Figure 6 Normalized expression levels of 17 top-ranked feature genes detected by EDGE for 293T and Jurkat cells. Genes are ordered by their importance scores with *HAND1* having the highest importance score.

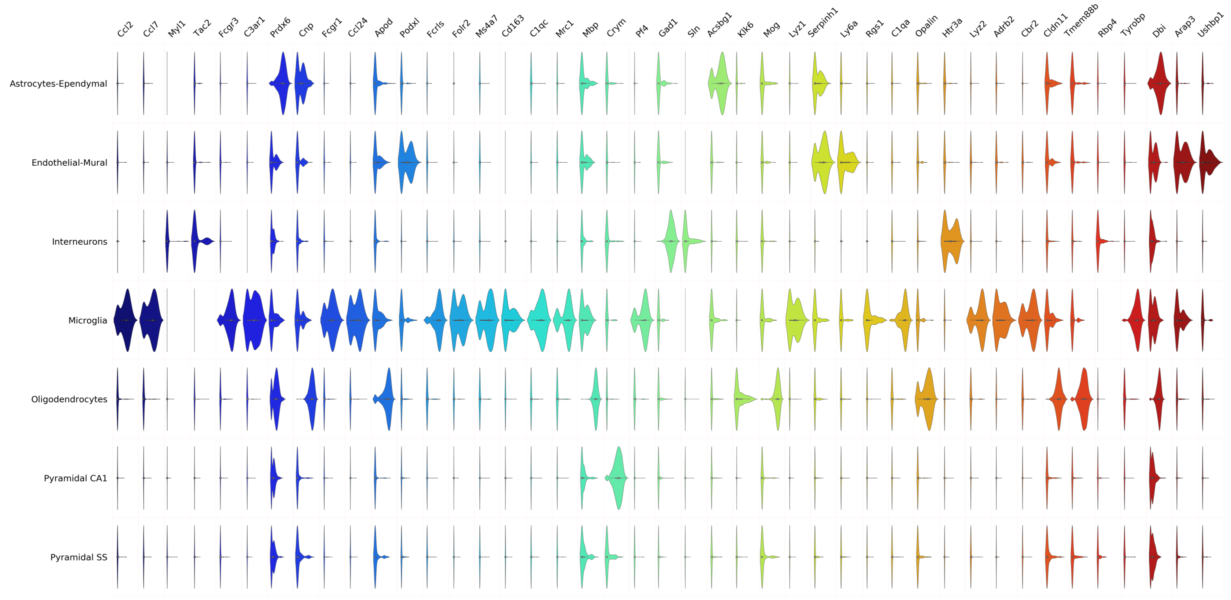

Supplementary Figure 7 Normalized expression levels of 43 top-ranked feature genes detected by EDGE for mouse brain dataset. Genes are ordered by their importance scores with *CCL2* having the highest importance score.

Feature genes identification in simulated datasets. There were two scenarios for the simulated datasets containing three cell types, with the ratio of 30:30:40 in group size. Since each cell type was associated with 15 genes, the number of true feature genes was 45 in the three-group scenarios. The results are reported in Supplementary Table 1. Among the top 15 genes selected by EDGE, the average number of true feature genes was 15.00 in the low dropout scenario and 14.94 in the high dropout one over 100 replications. Even in the top 45 genes selected by EDGE, our algorithm could identify 39.50 and 32.19 genes in the low and high dropout scenarios.

Supplementary Table 1 The performance of EDGE in detecting the feature genes in simulated studies with three cell types over 100 replications

	Top 15 Genes	Top 30 Genes	Top 45 Genes
Low Dropout	15.00 (0.00)	29.82 (0.46)	39.50 (1.80)
High Dropout	14.94 (0.24)	26.61 (1.67)	32.19 (2.37)

The standard deviation of the number of identified true feature genes is shown in the parentheses.

Supplementary discussion

From t-SNE and UMAP to EDGE. The dimensionality reduction methods convert the high-dimensional dataset $\mathbf{X} = \{\mathbf{x}_1, \cdots, \mathbf{x}_C\}$ into the low-dimensional dataset $\mathbf{Y} = \{\mathbf{y}_1, \cdots, \mathbf{y}_C\}$ [8]. In short, EDGE, t-SNE, and UMAP share the following procedures.

- Calculate the similarity probability p_{ij} for ith and jth cell, $i = 1, \cdots, C, j = 1, \cdots, C$, in the high-dimensional space based on the gene expression matrix $\mathbf{X}$.
- Estimate the similarity probability $q_{ij}, i = 1, \cdots, C, j = 1, \cdots, C$ in the low-dimensional space. The probability q_{ij} constructed by the embedding matrix $\mathbf{Y}$ matches p_{ij} as close as possible by minimizing a loss function.

The first step is critical in dimensionality reduction. If the similarity probabilities p_{ij} cannot preserve the similarity structures between cells faithfully, it is impossible to obtain an accurate similarity probability q_{ij}. One of our major contributions is proposing a novel method that is accurate in preserving the similarity structures in the high-dimensional space.

EDGE, t-SNE, and UMAP utilize three different ways to calculate p_{ij}'s in the first step. To compare these methods, we now present details of the calculation of similarity probabilities. In t-SNE, the conditional probability $p_{j|i}$ is calculated to represent the similarity of cell j to cell i. Mathematically, the conditional probability is defined by

$$p_{j|i} = \frac{\exp(-\|\mathbf{x}_i - \mathbf{x}_j\|^2 / 2\sigma_i^2)}{\sum_{k \neq i} \exp(-\|\mathbf{x}_i - \mathbf{x}_k\|^2) / 2\sigma_i^2}, \tag{1}$$

where σ_i is determined by the perplexity parameter [9]. The conditional probabilities in (1) can be symmetrized by defining the pairwise similarity $p_{ij} = \frac{p_{j|i} + p_{i|j}}{2C}$. In the UMAP algorithm, the following conditional probability is defined

$$p_{j|i} = \exp\left(\frac{-\max(0, d(\mathbf{x}_i, \mathbf{x}_j) - \rho_i)}{\sigma_i}\right), \tag{2}$$

where $d(\mathbf{x}_i, \mathbf{x}_j)$ is a distance measure, e.g., Euclidean distance, ρ_i is the distance from the ith cell to its first nearest neighbor, and σ_i is determined by the number of nearest neighbors k. In UMAP, a different symmetrization method for the conditional probabilities in (2) is considered, that is, $p_{ij} = p_{j|i} + p_{i|j} - p_{j|i}p_{i|j}$ [10]. Our similarity learning strategy is entirely different from the ones implemented in t-SNE and UMAP. We combine ensemble learning with the sketching technique to calculate similarity probabilities in the high-dimensional space. We designed a simulation study using Splatter to demonstrate the contributions of the first step in EDGE. To better present results graphically, we generated two cell types

with a ratio of 98:2 in the simulated data that contained 500 cells and 200 genes. The default parameters in Splatter were used. The similarity probabilities for EDGE, UMAP, and t-SNE were obtained in the high-dimensional space. We then applied the same embedding method

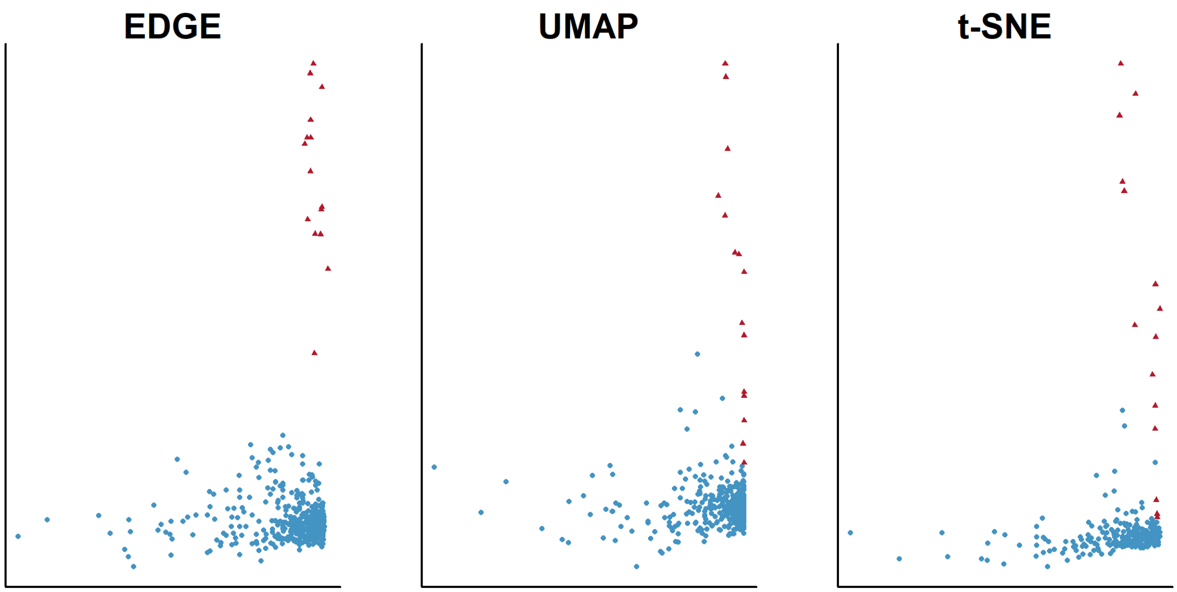

Supplementary Figure 8 Comparison of similarity learning strategies for EDGE, t-SNE and UMAP. Two types of cells are represented by red triangles and blue dots.

(spectral embedding) to map the data into a two-dimensional space based on three similarity matrices. For EDGE, rare and major cell types were well-separated; that was, the computed similarity matrix could preserve within- and between-cluster distances (Supplementary Figure 8). However, for embeddings constructed by UMAP and t-SNE, some rare and major cells were mixed (Supplementary Figure 8). To further verify this, we also replaced the similarity probabilities from UMAP and t-SNE with EDGE's similarity probabilities. If the EDGE's similarity probabilities were used in UMAP and t-SNE, rare and major cells were mapped to two different compact locations (Supplementary Figure 9). Nevertheless, based on the original similarity probabilities from UMAP and t-SNE, three to five rare and major cells were misclassified in the low-dimensional spaces. Therefore, our similarity learning algorithm has better performance in preserving within- and between-cluster similarities.

In the second step, t-SNE declares the Student's t-distribution for the distances (similarity probabilities) between the pairs of points $\mathbf{y}_i$ and $\mathbf{y}_j$, $i, j = 1, \cdots, C$, in the low-dimensional space. UMAP employs a similar distance distribution in the low-dimensional embedding, see details in Methods. We choose the same distance distribution used in UMAP for the low-dimensional embedding and the symmetrization method implemented in t-SNE as they

are computationally efficient. The loss function is critical in preserving the global structure.

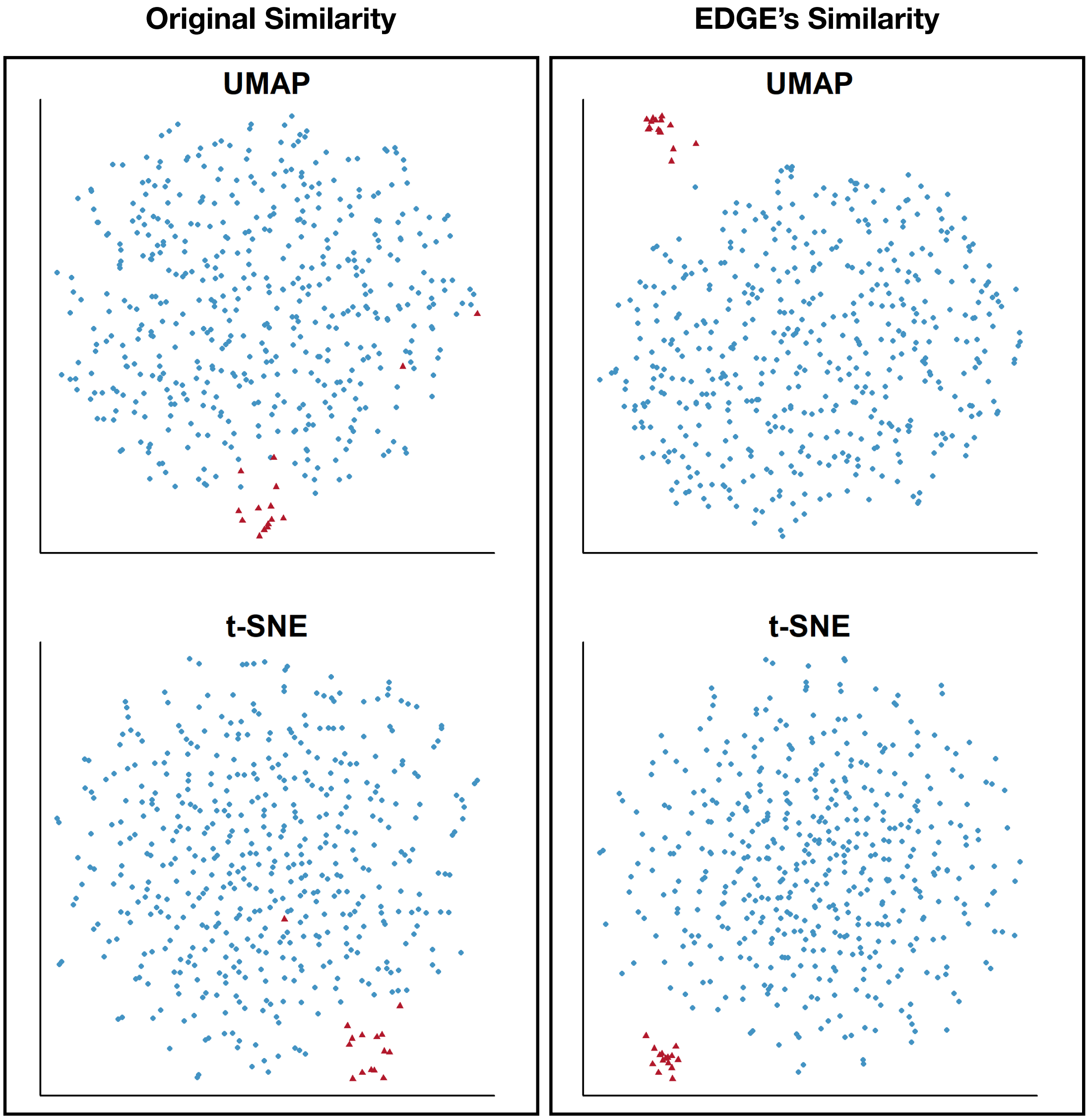

Supplementary Figure 9 Embedding results of UMAP and t-SNE by using the original (left panel) and EDGE's (right panel) similarity measures. Rare and major cells are represented by red triangles and blue dots.

Our goal is to project the high-dimensional probabilities onto the low-dimensional ones faithfully by minimizing the loss function, which measures the distance between the similarity distributions for low- and high-dimensional spaces. The t-SNE method implements the

Kullback-Leibler (KL) divergence loss function, which is defined as

$$\sum_i \sum_j p_{ij} \log\left(\frac{p_{ij}}{q_{ij}}\right). \tag{3}$$

The UMAP method uses binary cross-entropy (CE) as a loss function. Compared to (3), an additional term, $(1 - p_{ij})\log((1 - p_{ij})/(1 - q_{ij}))$, is present in the CE function. For an ideal case, we could have $p_{ij} = q_{ij}$ for all i and j. Under this situation, the values of KL and CE functions are zeros. In reality, it is unlikely to achieve such a perfect match. However, the probability p_{ij} in the high-dimensional space should be positively correlated with the probability q_{ij} in the low-dimensional space for all i and j. Thus, the value of loss function should be large if p_{ij} is large, while q_{ij} is low or vice versa. The additional term in the CE function is critical in preserving global distances. For instance, let $p_{ij} = 0.01$ and $q_{ij} = 0.99$, the value of the KL function is about zero, whereas, the value of the CE function is about 4.5. The CE function imposes a penalty for the case of low p_{ij} at large q_{ij}. We implement the CE function as a loss function in EDGE to preserve global distances.

In summary, in the first step, our novel similarity learning strategy can accurately preserve within- and between-cluster distances in the high-dimensional space. In the second step, computationally efficient techniques, i.e., the symmetrization method from t-SNE and the stochastic gradient descent algorithm, are implemented. To preserve global structures in the low-dimensional space, we employ the CE loss function in the second step.

Supplementary methods

Supplementary Algorithm 1: Similarity search

input: $\mathbf{X} \in \mathbb{R}^{C \times G}$

L: the number of weak learners

B: the number of genes to be randomly selected

H: the number of bins

$\mathbf{\Pi}[L][H][\cdot]$: store similar cells for different bins across weak learners

$\mathbf{\Gamma}[L][C]$: bin index

$\mathbf{S}[C][C]$: similarity matrix

$l \leftarrow min(\mathbf{X})$

$u \leftarrow max(\mathbf{X})$

$\mathbf{\Gamma}[L][C] \leftarrow [0][0]$

for $i = 1, ..., L$ **do**

  for $j = 1, ..., B$ **do**

    $\nu \leftarrow$ pick a random integer within $[1, G]$

    $\tau \leftarrow$ pick a random float within $[l, u]$

    $\omega \leftarrow$ pick a random integer

    for $k = 1, ..., C$ **do**

      $\beta \leftarrow 0$

      if $\mathbf{X}[k][\nu] \geq \tau$ **then**

        $\beta \leftarrow 1$

        $\mathbf{\Gamma}[i][k] \leftarrow \mathbf{\Gamma}[i][k] + \omega * \beta$

  for $k = 1, ..., C$ **do**

    $\mathbf{\Gamma}[i][k] \leftarrow \mathbf{\Gamma}[i][k] \% H$

    $\mathbf{\Pi}[i][\mathbf{\Gamma}[i][k]].append(k)$

$\mathbf{S}[C][C] \leftarrow [0][0]$

for $i = 1, ..., L$ **do**

  $\mathbf{S}_l[C][C] \leftarrow [0][0]$

  for $j = 1, ..., C$ **do**

    $\delta \leftarrow \mathbf{\Gamma}[i][j]$

    $\Delta \leftarrow \mathbf{\Pi}[i][\delta]$

    $K \leftarrow length(\Delta)$

    for $k = 1, ..., K$ **do**

      $\mathbf{S}_l[j][\Delta[k]] \leftarrow 1$

      $\mathbf{S}_l[\Delta[k]][j] \leftarrow 1$

  $\mathbf{S}[\cdot][\cdot] \leftarrow \mathbf{S}[\cdot][\cdot] + \frac{1}{L}\mathbf{S}_l[\cdot][\cdot]$

output: $\mathbf{S} \in \mathbb{R}^{C \times C}$

Supplementary Algorithm 2: Spectral embedding

input: $\mathbf{S}$,d
$\mathbf{D} \leftarrow$ degree matrix for $\mathbf{S}$
$\mathbf{A} \leftarrow \mathbf{D}^{1/2}(\mathbf{D} - \mathbf{S})\mathbf{D}^{1/2}$
$\mathbf{E} \leftarrow$ eigenvectors of $\mathbf{A}$
$\mathbf{E}_d \leftarrow$ top d eigenvectors from $\mathbf{E}$
output: $\mathbf{E}_d$

Supplementary algorithms. The details of the similarity search are shown in Supplementary Algorithm 1. Briefly, there are two major steps in the algorithm. In the first step, we implement the sketching technique to build hash codes. The hash codes are used to construct a similarity matrix of cells in the second step. We present details of spectral embedding in Supplementary Algorithm 2.

Hyperparameters selection. We illustrated the performance of EDGE under different parameter settings. As the number of weak learners increased, the pairwise distances between cells from the same cell type became smaller (Supplementary Figure 10). In the Jurkat dataset, EDGE separated the rare cells (Jurkat) from dominant cells (293T) when L reached 200 and maintained its performance as L continued to increase. Furthermore, EDGE was robust as the number of nearest neighbors

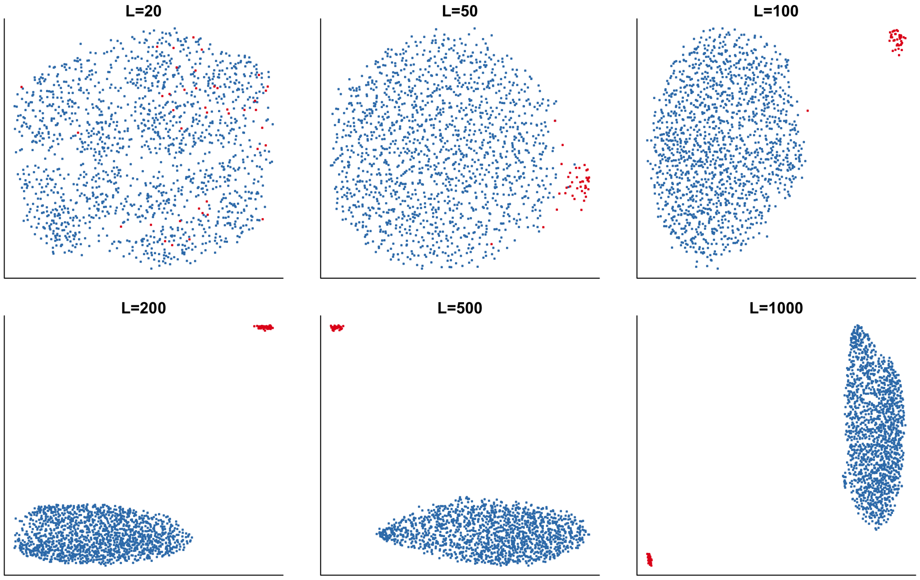

Supplementary Figure 10 The performance of EDGE in separating 293T and Jurkat (rare, in red) cells. The number of weak learners L is from 20 to 1000. Other parameters are fixed at $k = 20$ and $B = 15$.

varied from 10 to 50 (Supplementary Figure 11). In simulated datasets (Scenario 4 with DE gene proportion being 25%), EDGE maintained a high prediction accuracy with varying L, k, and B (Supplementary Figure 12). The computational time was also reported in the rightmost panel of Supplementary Figure 12. As L grew, the runtime increased but was no more than 15 seconds.

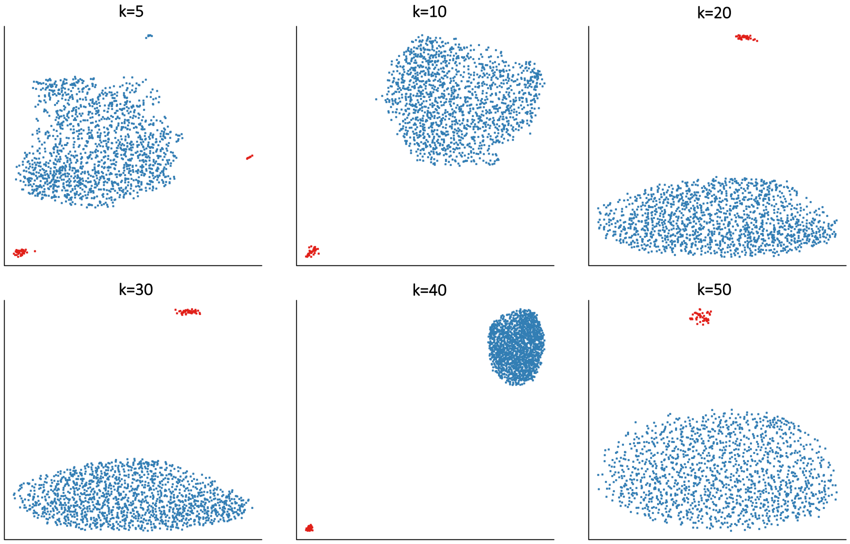

Supplementary Figure 11 The performance of EDGE in separating 293T and Jurkat (rare, in red) cells. The number of nearest neighbours k is from 5 to 50. Other parameters are fixed at $L = 200$ and $B = 15$.

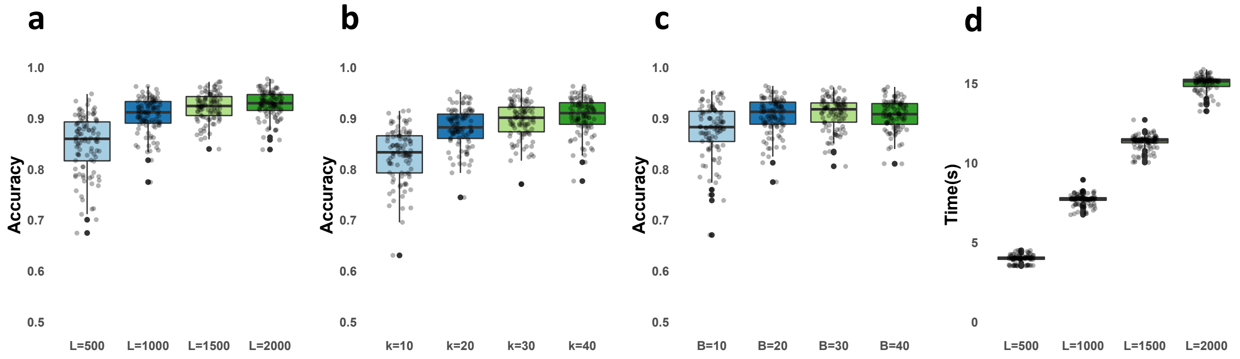

Supplementary Figure 12 The performance of EDGE for different hyperparameters in Scenario 4 of simulation studies. a-c: The prediction accuracy based on random forests for varying L, k, and B. d: The runtime for varying L. The results are summarized from 100 simulation runs. In the boxplots, we show the median (central lines), first and third quartile (box limits), and the whiskers extended to the lowest and highest points within 1.5 interquartile range of the first and third quartiles, respectively.

Supplementary References

[1] de Saint Basile, G. *et al.* Severe combined immunodeficiency caused by deficiency in either the δ or the ε subunit of CD3. *J. Clin. Invest.* **114**, 1512–1517 (2004).

[2] Li, J. *et al.* Interleukin-1β pre-treated bone marrow stromal cells alleviate neuropathic pain through CCL7-mediated inhibition of microglial activation in the spinal cord. *Sci. Rep.* **7**, 42260 (2017).

[3] Butovsky, O. *et al.* Identification of a unique TGF-β–dependent molecular and functional signature in microglia. *Nat. Neurosci.* **17**, 131-143 (2014).

[4] Zeisel, A. *et al.* Cell types in the mouse cortex and hippocampus revealed by single-cell RNA-seq. *Science* **347**, 1138–1142 (2015).

[5] Asuni, A. A. *et al.* Analysis of the hippocampal proteome in ME7 prion disease reveals a predominant astrocytic signature and highlights the brain-restricted production of clusterin in chronic neurodegeneration. *J. Biol. Chem.* **289**, 4532–4545 (2014).

[6] Yun, H.-M., Park, K.-R., Kim, E.-C. & Hong, J. T. PRDX6 controls multiple sclerosis by suppressing inflammation and blood brain barrier disruption. *Oncotarget* **6**, 20875–20884 (2015).

[7] Haidet-Phillips, A. M. *et al.* Astrocytes from familial and sporadic ALS patients are toxic to motor neurons. *Nat. Biotechnol.* **29**, 824–828 (2011).

[8] Tenenbaum, J. B., De Silva, V. & Langford, J. C. A global geometric framework for nonlinear dimensionality reduction. *Science* **290**, 2319–2323 (2000).

[9] Maaten, L. v. d. & Hinton, G. Visualizing data using t-SNE. *J. Mach. Learn. Res.* **9**, 2579–2605 (2008).

[10] McInnes, L., Healy, J. & Melville, J. UMAP: uniform manifold approximation and projection for dimension reduction. Preprint at `https://arxiv.org/abs/1802.03426` (2018).
