# Supplementary material for: Ensemble dimensionality reduction and feature gene extraction for single-cell RNA-seq data
Source: Nat Commun. 2020 Nov 17;11:5853. doi: 10.1038/s41467-020-19465-7 (PMC7673125; doi:10.1038/s41467-020-19465-7)
Supplement: Supplementary file 3 — Descriptions of Additional Supplementary Files [file 41467_2020_19465_MOESM3_ESM.pdf]

Descriptions of Additional Supplementary Files

Supplementary Data 1

Description: Identified feature genes for three scRNA-seq datasets
